# Moving ecological and biogeochemical transitions across the North Pacific

**DOI:** 10.1002/lno.11763

**Published:** 2021-05-05

**Authors:** Christopher L. Follett, Stephanie Dutkiewicz, Gael Forget, B. B. Cael, Michael J. Follows

**Affiliations:** ^1^ Department of Earth, Atmospheric and Planetary Sciences Massachusetts Institute of Technology Cambridge Massachusetts USA; ^2^ National Oceanography Centre Southampton UK

## Abstract

In the North Pacific Ocean, nutrient rich surface waters flow south from the subpolar gyre through a transitional region and into the subtropics. Along the way, nutrients are used, recycled, and exported, leading to lower biomass and a commensurate change in ecosystem structure moving southward. We focus on the region between the two gyres (the Transition Zone) using a coupled biophysical ocean model, remote sensing, floats, and cruise data to explore the nature of the physical, biogeochemical, and ecological fields in this region. Nonlinear interactions between biological processes and the meridional gradient in nutrient supply lead to sharp shifts across this zone. These transitions between a southern region with more uniform biological and biogeochemical properties and steep meridional gradients to the north are diagnosed from extrema in the first derivative of the properties with latitude. Some transitions like that for chlorophyll *a* (the transition zone chlorophyll front [TZCF]) experience large seasonal excursions while the location of the transitions in other properties moves very little. The seasonal shifts are not caused by changes in the horizontal flow field, but rather by the interaction of seasonal, depth related, forcing with the mean latitudinal gradients. Focusing on the TZCF as a case study, we express its phase velocity in terms of vertical nutrient flux and internal ecosystem processes, demonstrating their nearly equal influence on its motion. This framework of propagating biogeochemical transitions can be systematically expanded to better understand the processes that structure ecosystems and biogeochemistry in the North Pacific and beyond.

Here we consider the features and dynamics of the Transition Zone between the subpolar and subtropical gyres in the North Eastern Pacific. Macronutrients are supplied by wind‐driven upwelling to the surface subpolar gyre, fueling vigorous, seasonal production. In contrast, the downwelling subtropical gyre is macronutrient‐limited. The subpolar gyre is characterized by communities with a high abundance of larger primary producers (Bogorov 1958) while productivity in the subtropical gyre is dominated by pico‐phytoplankton. Between the gyres, approximately 30°–45°N, is a zone characterized by a number of sharp ecological and biogeochemical transitions (Fig. 1) (Polovina et al. 2000; Church et al. 2008), likely determined by the interplay of the regional ecology, chemistry, and physics.

**Fig 1:**
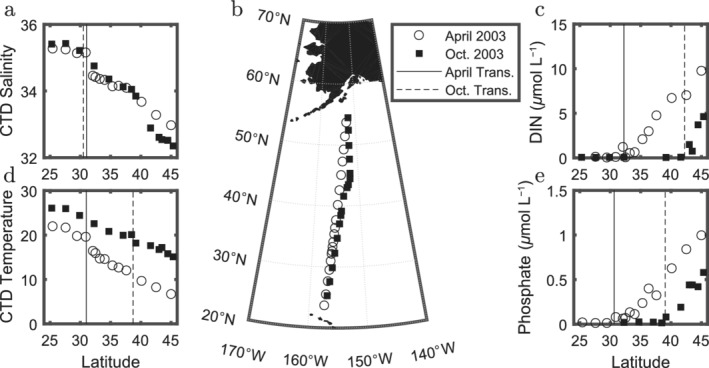
Static and moving transitions are seen in observations from two cruises, panel (**b**), between Hawaii and Alaska (Church et al. 2008) in 2003, open circles for April and black squares for October. Shown against latitude are surface (**a**) salinity (psu); (**c**) DIN (*μ*mol L^−1^); (**d**) temperature (°C) and (**e**) phosphate (*μ*mol L^−1^). Solid vertical lines indicate the location of the transition in each field for October and dashed for April. Transitions are defined by the maximum in the latitudinal derivative for the field (for salinity, temperature) or the logarithm of the field (for DIN and phosphate). Data can be found at https://hahana.soest.hawaii.edu/cookbook/cookbook.html.

The most studied expression of this interplay is the location of the transition zone chlorophyll front (TZCF; Polovina et al. 2001; Bograd et al. 2004; Polovina et al. 2017), often operationally defined by the 0.2 *μ*g L^−1^ chlorophyll contour (Fig. 2) (Polovina et al. 2001). This boundary is associated with a number of migrating predators in the region (Polovina et al. 2001) including tuna, loggerhead turtles, and squid. Thus, the TZCF is both an important ecological and economic demarcation in the Northern Pacific.

**Fig 2:**
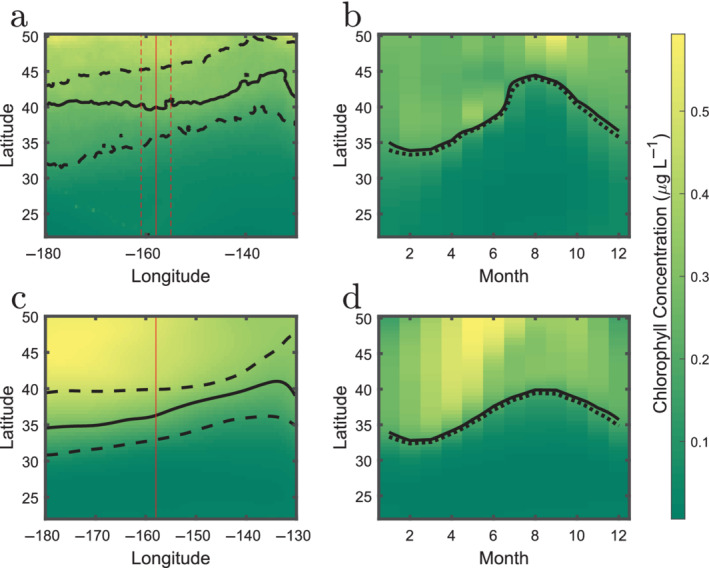
The TZCF is observed in satellite products and model output. Left column (**a**, **c**) shows maps in the North Pacific (longitude/latitude) of annual climatology of surface Chl *a* concentrations (*μ*g Chl L^−1^) from (**a**) satellite, and (**c**) model. Solid black lines indicate the 0.2 *μ*g L^−1^ contour, dashed lines indicate the maximal seasonal extent of this contour. Red vertical line indicates a N/S transect along 158 W from which data is shown in panels (**b**, **d**), and in subsequent plots. (**a**, **b**) For the satellite output, we used data from a range of longitude for the transect plot (indicated by the vertical dashed lines). Panels (**b**, **d**) show the transect as a function of month, top from satellite, bottom for model output. The solid black line again indicates the 0.2 *μ*g L^−1^ contour. The dashed black line indicates the location of the transition in Chl *a* as determined by the derivative method discussed in the text.

The latitudinal position of the TZCF progresses over the seasons (Fig. 2; Polovina et al. 2001; Bograd et al. 2004; Polovina et al. 2017), shifting by approximately 10° latitude over the course of the year. The southernmost seasonal extent of the TZCF occurs in very early springtime, when the mixed layer begins to shoal but surface nutrient concentrations are still maximal (Glover et al. 1994). As the season progresses, the front moves northward: driven by the interaction between the repartitioning of nutrients from inorganic to organic forms, particle export, and the strong northward increase in macronutrient supply (Glover et al. 1994; Bograd et al. 2004). The rate of southward progression of the TZCF in fall/winter has been attributed to southward advection (Ayers and Lozier 2010) or the progression of vertical destratification (Glover et al. 1994; Bograd et al. 2004).

In addition to the highly visible TZCF, the North Pacific Ocean has several additional eco‐chemical transitions. April and October cruises in 2003 (Church et al. 2008) show clear transitions in a number of physical, nutrient, and ecological properties (*see* Fig. 1). However, these transitions are typically not coincident with one another and do not move in concert. Hence, they are not all simply controlled by the same mechanisms. As an example, both surface dissolved inorganic nitrogen (DIN) and phosphate are present at extremely low concentrations across the subtropical gyre, and then, in the vicinity of the Transition Zone, change to exhibit a strong poleward increase. These two transitions are both displaced by hundreds of kilometers between April and October cruises but they are decoupled and their relative distance also increases by 200 km. Since they do not move in concert, it seems unlikely that they can be tied to a single physical (or biogeochemical) mechanism. We seek to build a consistent synthesis and general interpretation of these several transitions and their seasonal excursions. By understanding the contrasting patterns, we can reveal the relative importance of physical, biogeochemical, and ecological drivers in the system.

In this article, we first define a standard algorithm to define any such transition. Typically, the TZCF, for example, has been defined by a specific value of the chlorophyll concentration (Polovina et al. 2000). Here, to contrast and interpret the differential movement of several transitions, we systematically define them as concentrations indicative of places in space and time where the gradient of the surface concentration of a tracer changes sharply. We then define multiple transitions in the context of a physical‐biogeochemical model. Ecosystem relevant quantities have transitional boundaries which are characterized by two extremes: a large, seasonal north–south excursion as expressed by the TCZF and temperature; or a relatively static transition like the southern salinity front which we suggest defines the gyre boundary. We then show that both satellite and the ship‐based data are consistent with the model analysis. Finally, returning to the TZCF, we show how the phase velocity of the transition can be expressed mathematically, and modeled in terms of both vertical and internal ecosystem processes.

## Methods

We develop a method to determine the transitions of any property in a nonarbitrary manner. We do so first using output from numerical simulations (Dutkiewicz et al. 2015; Forget et al. 2015). Numerical simulations not only provide access to many fields, but the simulated data are sampled continuously. This generates smooth fields which greatly simplifies the method for determining the transition locations. Having developed the method using simulated data, we apply it to an analysis of transitions from satellite remote‐sensing products, float, and shipboard data. We briefly outline the different data sources and products (providing more detailed descriptions in the Supplemental Information) before we describe the methodology for determining the transitions.

### Fields used for analysis of the transitions

#### Physical/biogeochemical model

This model includes data‐contrained physical circulation and mixing (Forget et al. 2015) at 1° horizontal resolution coupled to a biogeochemical/ecosystem model (Dutkiewicz et al. 2015). The latter component captures the cycling of C, N, P, Si, and Fe as they pass through inorganic and (dead and living) organic pools. The living pools include a number of plankton functional groups, including cyanobacteria, diatoms, and zooplankton. We use surface (0–10 m) 20 yr (1992–2011) monthly climatologies of both physical and biogeochemical output projected onto a 0.5 × 0.5° grid. The latitudinal distribution and seasonality of bulk ecosystem properties such as chlorophyll (*see* Fig. 2c,d), phytoplankton biomass, nutrient concentrations, as well as the distributions of functional groups are plausible in comparison with satellite and in situ observations (Kuhn et al. 2019; Dutkiewicz et al. 2020; Sonnewald et al. 2020). For more details, see Supplemental Text S1. The range of physical, chemical, and biological variables within the model outputs allows us to examine the contrasting trajectories of different types of transitions.

#### Satellite products

We use monthly “climatologies” of several satellite products that are regridded to the same 0.5 × 0.5° grid as the model output. We use sea surface salinity (SSS) from SMAP (Fore et al. 2016), sea surface temperature (SST) data from GHRSST Level 4 AVHRR_OI Global Blended Sea Surface Temperature Analysis (GDS version 2) (Banzon et al. 2016), and chlorophyll (Fig. 2a,b) from GlobColour product (Garnesson et al. 2019). For more details, see Supplemental Text S2.

#### Float products

We use a recently published climatology from GPS tagged surface drifters (Laurindo et al. 2017) for the surface velocity field.

#### In situ data

We use data collected on the two cruises taken in 2003 (April and October) between Hawaii and Alaska (Church et al. 2008) shown in Fig. 1. We consider several physical (SSS, SST) as well as biogeochemical fields such as nutrients and chlorophyll *a* (Chl *a*).

### Determining transition boundaries

Here we seek to compare transitions across variables, and so must define an objective method which can be applied to any tracer, *c*, measured along a meridional transect. We do so by characterizing change points in the meridional gradients of the tracers.

For continuous sections of the tracer surface where the tracer gradient (*dc*/*dy*) has the same sign, we consider contours of *c*(*y*, *t*), where *y* and *t* represent latitude and time, respectively. We seek tracer contours whose latitude can be written as a function of time, *y*_*c*_(*t*), and are single‐valued over the entire time interval. From this space of contours, we choose the tracer contour which maximizes, *f*(*c*), the average value of the first derivative over time,

(1) $$f\left(c\right)=\frac{1}{t_{\max}-t_{\min}}\int_{t_{\min}}^{t_{\max}}\left|\frac{\partial c\left(y_{c},t\right)}{\partial y}\right|\mathrm{dt}.$$

In some cases, multiple extreme values could be chosen which represent different transitional boundaries. Higher or lower order derivatives could also be chosen. The specific implementation of this general approach will vary depending on the data available. The primary data sources in this work are gridded climatological model products which are smooth and monotonic inside the Transition Zone. They form surfaces in month and latitude space on which we generate 30 evenly spaced contours in tracer space. We then estimate the spatial derivative from the average latitudinal distance between neighboring contours. This operation can be done in either linear or log‐space depending on the nature of the tracer in question.

**Fig 3:**
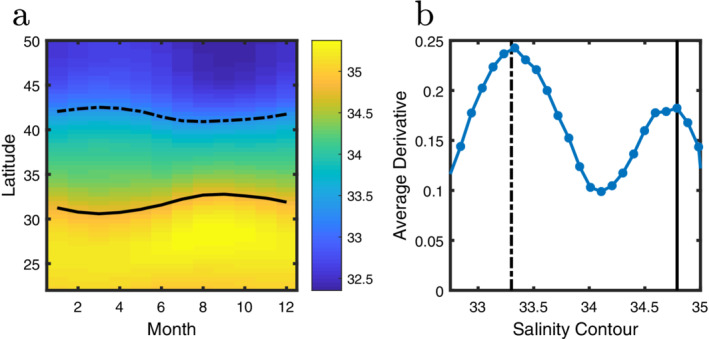
Northern (dashed black line) and southern (black line) boundaries of the transition zone can be determined from salinity transects. (**a**) Model salinity (colored shading, psu) along the 158 W transect (shown in Fig. 2) as a function of month. Overlaid are the northern (dashed) and southern (solid) black salinity contours used to estimate the extent of the transition zone. (**b**) The first derivative (psu deg.^−1^) of the annual average salinity with respect to latitude, plotted as a function of salinity contours. The two maxima of the derivative are used to define a northern (dashed black line) and southern (solid black line) transition in salinity and correspond to the contours in (**a**). Many of the other fields studied only have one transition in this region.

We confine our investigation to the Transition Zone, and use changes in the latitudinal salinity field to define its southern and northern boundaries; the Subtropical Convergence Zone and the Subarctic Convergence Zone, respectively (Roden 1991). Figure 3 shows climatological, monthly, mixed‐layer salinity from the model, along with the average first spatial derivative of the salinity field as a function of contour value. We define the Transition Zone boundaries as the two maxima in the spatial derivative. This exercise can be repeated with any available field (or property) to locate and track transitions from latitudinal transects and track their movements over time. We illustrate the transition of modeled and remotely sensed Chl *a* found using this method in Fig. 2. As shown in the figure, the method chooses a location for the time evolving Chl *a* front that is located very close to the 0.2 *μ*g L^−1^ contour used in prior work (Polovina et al. 2000, 2017). We believe that deviations between the model and satellite chlorophyll fields north of the Transition Zone are due to gaps in our understanding of iron limitation at high latitudes (Tagliabue et al. 2016). We will quantify the differences between the TZCF trajectories in the final section.

We use monthly gridded climatological data for both the model and satellite fields, but this is not possible when using individual transects from shipboard measurements. For use with the previously published cruise data (Church et al. 2008), see Figs. 1, 5c, the method must be modified to handle mesoscale fluctuations and noise. We first reduce the analysis to the latitudinal region of interest, in this case 25–45°N, to satisfy the monotonicity requirement discussed above. We segment the tracer space into *j* = 1 − 100, evenly spaced levels and use a linear interpolation *C*_*k*_(*y*) to find the set of latitudes *y*_*i*_(*c*_*j*_) which our data suggest have that tracer value. In this case, *k* = 1, 2 is the cruise and *i* is the intersection of the contour with the interpolant. The noise in the data is what causes multiple intersections per contour level. We use a linear interpolant to estimate the average spatial derivative for each level,

(2) $$f_{k}\left(c_{j}\right)=\frac{1}{n}\sum_{i=1}^{n}\frac{{\mathrm{dC}}_{k}\left(y_{i}\left(c_{j}\right)\right)}{\mathrm{dy}},$$

and then find the transition contour, (*j* ^*^), by maximizing

(3) $$f\left(c_{j}\right)=\frac{1}{2}\sum_{k=1}^{2}f_{k}\left(c_{j}\right).$$

The latitudinal location for this transition is then the average of the crossings $\langle y_{i}\left(c_{j^{*}}\right)\rangle$.

## Results and discussion

### Observed transitions

**Fig 4:**
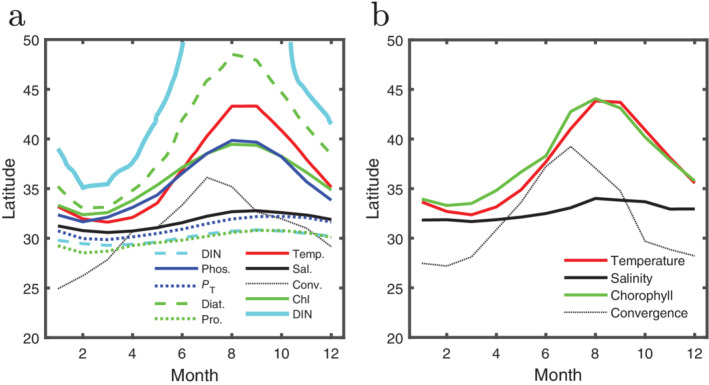
Latitudinal locations of the transitions for (**a**) model output and (**b**) satellite/float derived products along 158 W transect (shown in Fig. 2) as a function of month. We show transitions in physical fields (e.g., temperature, salinity, and convergence) as well as several biogeochemical and ecological fields (Chl, nutrients, and phytoplankton functional types). Most fields have only a single transition in this region, DIN and salinity two have two. For clarity, we only show the southern salinity boundary.

Using the above methods combined with the output from the biogeochemical/ecosystem model, we define a set of transitions for our transect over time. Transitions include both physical (temperature [SST], meridional/latitudinal velocity [*v*], salinity [*S*]) and biogeochemical (chlorophyll; DIN; phosphate; total phosphorus [*P*_T_]; relative abundance: diatoms and prokaryotes) transitions. We broke from our normal methods for two transitions. For meridional velocity, we choose the convergence transition (meridional velocity, *v* = 0) because of the strong physical implications for this boundary. For DIN, our normal method generates the southern (dashed, cyan) DIN transition in Fig. 4. There are large temporal changes in DIN at high latitudes which prevent a continuous northern transition contour emerging. We thus allow the algorithm to consider contours which are not continuous and find a second transition to the north (solid cyan in Fig. 4).

Considering the physical fields only, we note that salinity, which has a long response time to seasonal forcing, has a weak latitudinal excursion. On the other hand, SST and convergence have large excursions. These fields are strongly affected by seasonal forcing. For temperature, increased insolation and shoaling of the mixed layer lead to a fast response. The wind field, which drives the circulation, is also thermally forced, which leads to the large excursion in the convergence line.

The responses of the three physical fields in the model capture those found from the analogous satellite/float fields (Fig. 4). The equivalence of the salinity and temperature transitions is to be expected as the ECCOv4 model assimilates this data as part of the state estimate (Forget et al. 2015).

The other transitions shown in Fig. 4 are either biological or biogeochemical. Chlorophyll (TZCF), the relative abundance of diatoms, phosphate, and DIN (northern transition) all have large seasonal excursions in location. This behavior is consistent with the seasonal excursion found in satellite derived chlorophyll (Fig. 4) and the nutrient transects from Fig. 1. Transitions in the relative abundance of prokaryotes, the total integrated phosphorus, and DIN (southern transition) show weak seasonal movement. Total integrated phosphorus, *P*_T_, is the sum of all phosphorus atoms at a location, independent of speciation, and including both dissolved and particulate forms. We use *P*_T_ because biological repartitioning does not change its concentration.

The behavior of the transitions is quite different depending on which variable we consider. Some transitions embark on large seasonal excursions. The temperature transition, for example, moves almost 10° meridionally over the course of the year. On the other hand, the southern salinity transition has at most a 2° meridional variation. The biogeochemical transitions mostly exhibit some in‐between range of location shifts.

**Fig 5:**
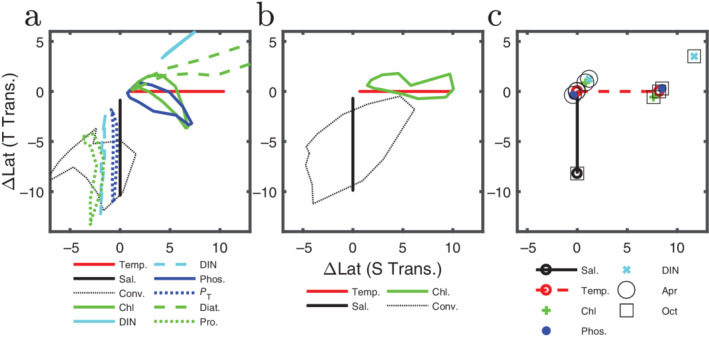
The location of each transition is plotted relative to the more static (southern) salinity transition (black line in Fig. 4) and the more seasonally varying location of the temperature transition (red lines in Fig. 4). Relative location is calculated for each month as the number of degrees of latitude between the transition of interest (e.g., Chl) and the (southern) salinity transition (horizontal axis, ΔLat [S transition]) and from the temperature transition (vertical axis, ΔLat [T transition]). These differences are plotted for each month, giving an indication of the trajectory of the seasonal cycle in this phase space. Some transitions are more static (shape of trajectory is more vertical) or more seasonally varying in location (more horizontal), but many have both components (more circular). (**c**) Cruise data (Church et al. 2008) plotted similar to panels (**a**) and (**b**). Circles are for the April 2003 cruise, and squares for the October 2003 cruise. Black: salinity; red: temperature; green: Chl *a*; dark blue: phosphate; light blue: nitrite + nitrate.

One way to explore the relationships between these fields is to plot the location of the transitions across the seasonal cycle relative to physical transitions. In Fig. 5, we plot, for each month, the location of the transitions relative to the location of the southern salinity transition (southern boundary of the Transition Zone) along the x‐axis and relative to the location of the temperature transition along the y‐axis. The axes then show the number of degrees latitude between the location of the transition of the property of interest (e.g., Chl) and the salinity (*x*) and temperature (*y*). In this space, the salinity transition (black) follows a vertical trajectory, while the temperature transition (red) follows a horizontal one. Plotting the locations of the biological response variables against the temperature and salinity transition makes it clear that there are two classes of physical transitions and that most biogeochemical transitions behave like a mixture of the two. The salinity and temperature transitions represent the physical extremes: the salinity transition is effected primarily by water mass flow, and temperature is driven by seasonal heating. Vertical trajectories (which follow the salinity transition's movement) in Fig. 5 thus represent relatively steady processes whereas horizontal trajectories (which follow the more extreme movement of the temperature transition) are ones which respond strongly to seasonal forcing. A combination of external forcing and internal controls determines the migration of a transition leading many to exhibit in‐between trajectories.

This behavior is also visible in the field transects (Church et al. 2008) (*see* Fig. 1). In Fig. 5c, we plot the results from the two transects in the same space as in Fig. 5a,b. The scattered points from April and October of the same year are consistent with the model and satellite data. In April, the transitions are all close together, but are far more separated by October (~ 2° vs. ~ 4° for the biogeochemical transitions). Panel c shows that cruise data provide only a snapshot of the rapidly shifting transitions. Phase diagrams such as this one can help place cruise data within a larger temporal scale context. The degree to which the transition of a given variable migrates seasonally or remains approximately steady, can be seen by the trajectory it traces in this space: a horizontal trajectory shows strong seasonal variability, similar to the temperature transition; a vertical trajectory means small seasonal variability, similar to salinity; and a circular trajectory is somewhere in between. DIN, phosphate, and chlorophyll are all highly seasonal as seen by the horizontal (two point) trajectories in Fig. 5c. We might imagine that similar processes, like growth and export, drive both nutrient and chlorophyll transitions northward between spring and summertime. Additional information comes from the dispersion between the seasonal transitions.

### Genesis of transitions and their seasonal motion

**Fig 6:**
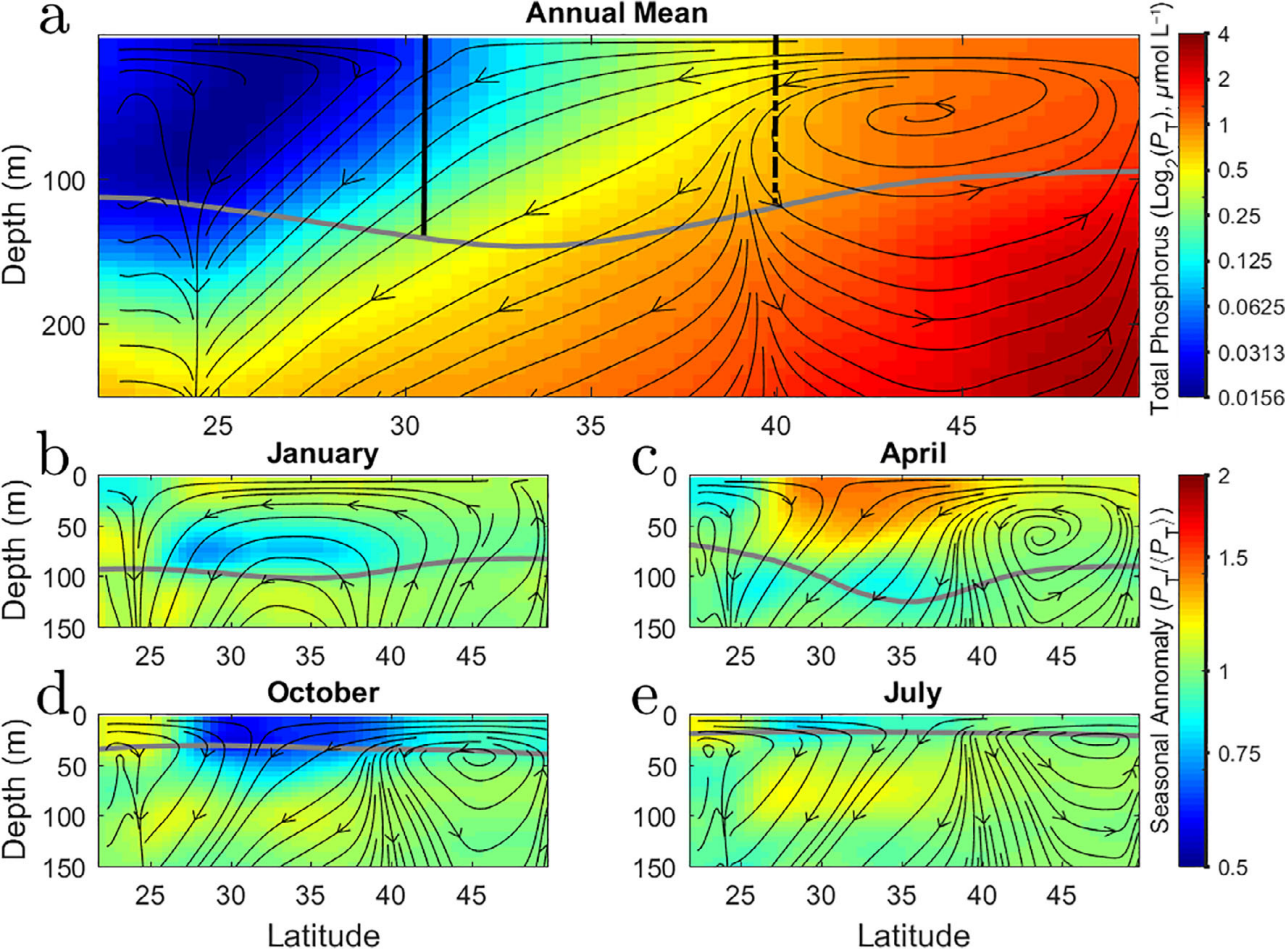
Modeled latitude‐depth transects along 158 W. (**a**) Shading is the annual mean model total phosphorus concentration (log_2_ *μ*mol L^−1^), *P*_T_, which includes all inorganic and organic forms of phosphorus. Lines with arrows indicate the streamlines as determine from the vertical and meridional velocities. The gray line indicates the mixed layer depth. Vertical solid black and dashed lines in are the approximate locations of the southern and northern boundaries of the transition zone shown using salinity in Fig. 3. (**b**–**e**) Shading is the seasonal anomaly of total phosphorus concentration (unitless) presented as the seasonal mean divided by the annual mean and presented in log space. Positive (redder) indicates higher than annual average and negative (bluer) indicates seasonal average is lower. Arrows and lines as in (**a**) but for specific months. (**b**) Janaury (winter), (**c**) April (spring), (**d**) October (fall), (**e**) July (summer).

**Fig 7:**
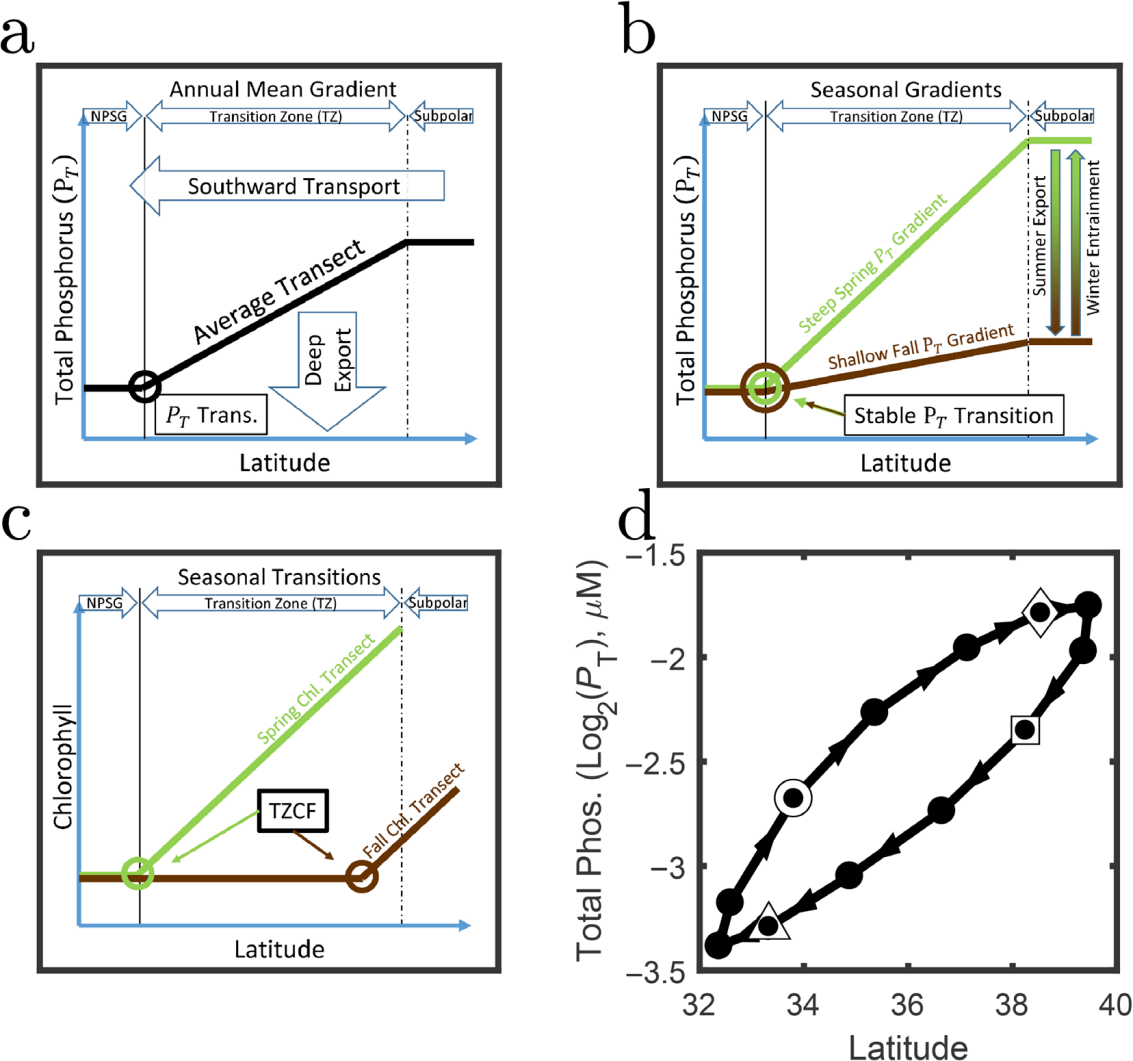
Schematics of mechanisms behind transition formation and seasonal migration. (**a**) The annual average gradient in total phosphorus (which can be thought of as approximately following nutrient supply) is set by a balance between the southward transport and the gradual export of material below the mixed layer. (**b**) Seasonal changes in the slope of the total phosphorus (*P*_T_) are caused by seasonal export and entrainment due to oscillations in the mixed layer depth. The gradient in *P*_T_ changes but the location of the transition stays relatively fixed. The changing gradient leads the meridional location of some transitions to move. (**c**) Seasonal forcing leads some transitions like the TZCF to move considerably over the season. (**d**) The trajectory of the chlorophyll transition, TZCF, over latitude relative to total phosphorus. Solid black circles denote each month. White markers denote January (triangle), April (circle), July (diamond), and October (square).

The balance between the vertical export of organic particles and the southward flow of nutrients generates the background gradient in nutrient supply which causes the biogeochemical transitions. Figure 6a shows the model flowlines for the annual climatology overlayed on the concentration of total phosphorus. The flowlines lay out the vertical structure of the intergyre system (*see* Fig. S1 and Supplement for a more detailed discussion of the flow). North of the Transition Zone (vertical dashed line) water with high total phosphorus concentration is upwelled into the mixed layer (gray curve). This water flows into the Transition Zone where it continues to move southward at approximately 3° per year. Along the way, phosphorus is lost through the export of particles and subduction below the mixed layer. The approximate southern edge of the Transition Zone (vertical black line) aligns with flow convergence in the very surface, but the bulk surface flow is never‐the‐less southward when averaged over the mixed layer. Nutrients thus continue to flow southward into the North Pacific Subtropical Gyre. Horizontal transport into subtropical gyres from high latitudes is critical for maintaining primary production in these regions (Williams and Follows 2003; Palter et al. 2011; Yamamoto et al. 2018). The balance between the southward advection and vertical export, shown schematically in Fig. 7a, leads to the monotonic gradients in total phosphorus (equated to nutrient supply) between the gyres. Biological interactions with this background nutrient supply gradient, modified by light and temperature, lead to the formation of the biogeochemical transitions.

Sharp transitions in the biogeochemical tracer concentrations occur because the growth rates and interactions between phytoplankton groups depend nonlinearly on the available nutrients. A manifestation of this can be seen in the occurrence of large phytoplankton when compared to small cyanobacteria (Dutkiewicz et al. 2009; Follows et al. 2018). Under low nutrient supply rates, small cells (with higher surface area to volume ratios) are selected for over larger cells. Increasing the supply rates increases the population of small cells while keeping the ecosystem composition relatively constant. However, because predation and viral mortality scale with the square of the population, small phytoplankton can only increase in abundance to a certain level before larger cells appear (Armstrong 1994; Poulin and Franks 2010). This theory is used to mathematically explain the transition from a cyanobacteria dominated ecosystem in the gyres to an ecosystem dominated by larger cells at high latitudes (Ward et al. 2014). These phytoplankton compositional shifts should be accompanied by shifts in the biologically available nutrients (e.g., phosphate). These shifts can be seen in both the transitions from the model output and those from the two COOK‐BOOK cruises (Figs. 1, 5) (Church et al. 2008).

Seasonal changes in the location of transitions are caused by modifications to the slope of the background nutrient, light, and temperature gradients. The schematic in Fig. 7b shows how the gradient in total phosphorus decreases with the summertime export of organic particles and increases as the mixed layer deepens and entrains remineralized nutrients. The modeled (and real ocean) mixed layer depth has a seasonal fluctuation of approximately 100 m which directly affects *P*_T_ in the surface. Such deviations from the background gradient in model *P*_T_ over the transect are shown across the seasons in Fig. 6b–e. Warm colors represent positive, logarithmic (Log_2_), deviations from the local annual mean and cool colors are negative deviations. Starting in January, a deepening mixed layer brings *P*_T_ from depth to the surface. This leads to positive deviations at the surface (0–50 m) and negative deviations deeper down (50–100 m), but still within the mixed layer. This effect is seen even more strongly in April. By July, sinking and remineralizing particles have moved *P*_T_ to deeper depths (positive anomaly at 50–100 m). In October, the effect of vertical transport from sinking particulates is expressed clearly in the negative anomalies in the surface 0–50 m. Higher winds and cooling then further deepen the mixed layer entraining nutrients which bring the transitions to their southernmost location around March.

Differing rates of repartitioning and seasonal succession lead biogeochemical transitions to move at different speeds causing them to disperse as they move north in the spring/summer and coalesce again moving southward in the fall/winter. The coherent change in the slope of the background gradients in light, temperature, and total nutrient over the season drive the motion of all the biogeochemical transitions. Their motion relative to one another (Fig. 4 and the differently shaped trajectories in Fig. 5) is a quantitative expression of biogeochemical processes.

One way to think about this is to follow a transition as it progresses meridionally over the year. Figure 7c shows a schematic of the seasonal progression of the TZCF. One explanation for this motion is the changing meridional gradient in *P*_T_ over the season. If the chlorophyll concentration at the transition was set entirely by *P*_T_, we would expect the TZCF to move meridionally with the *P*_T_ gradient. In Fig. 7d, we plot the seasonal trajectory of the latitude of the transition in chlorophyll (TZCF), vs. total phosphorus, *P*_T_. If the partitioning of chlorophyll was indeed a constant fraction of nutrient supply, we would expect the trajectory to be horizontal. A single value of *P*_T_ would align with the TZCF. The elliptical trajectory in Fig. 7d suggests that variations in *P*_T_ can only explain about half of the excursion. The remainder (i.e., variations causing the trajectory to deviate from horizontal) must be due to other biological and ecological processes. For nutrients like phosphate and DIN, a similar approach could be taken using differences in the relative speeds of their transitions to quantify the relative turnover times between inorganic and organic nutrients.

Seasonal mixing, particle dynamics, and the ecosystem response all work together with the latitudinal gradients to generate a pattern of seasonally propagating transitions in the North Pacific. Variables which are governed only by the average flow field have horizontal contours, and hence transitions, which stay relatively fixed in latitude. Variables which respond strongly to seasonal forcing have contours with greater slopes, and hence transitions which move further with season. In the North Pacific, the time independent transitions tend to follow the southern salinity transition. Strongly seasonal variables tend to have transitions that more closely follow the temperature transition. Among the modeled biogeochemical variables, the relative abundance of prokaryotes undergoes a sharp transition near the southern salinity front, along with the transition in total phosphorus, *P*_T_, and DIN (southern transition). The transition in *P*_T_ corresponds to a shift in nutrient supply, with a rapid increase in *P*_T_ and nutrient supply moving northward. The seasonal forcing in the subtropical gyre south of the Transition Zone is never large enough for larger cells to compete with small prokaryotes, but this changes near the southern boundary of the Transition Zone, where seasonality starts to effect composition. Larger cells (including diatoms) grow less efficiently at low nutrient concentrations and are mostly excluded from the gyre. They emerge north of the *P*_T_ transition and are far more seasonally controlled. Thus, the diatom boundary efficiently tracks and extends past the temperature/light forcing and its seasonal latitudinal oscillation. South of the Transition Zone nutrients are mostly tied up in the organic phase. As nutrients increase to the north, there is a demarcation between a weakly seasonal and highly seasonal regime. North of the southern Transition Zone boundary, when biogeochemical transitions exist, they tend to move seasonally.

### Connecting transition dynamics to mechanistic drivers

The temporal movements of the transitions are driven by the physical and biogeochemical forcing. By examining these trajectories, we can begin to understand the role of different types of forcing. The observed phase velocity (latitudinal shift) of the transition of any field, *c*, *dy*_*c*_/*dt*, can be expressed as a sum of the phase velocity due only to forcings and the flow transport velocity, *v*, of that field:

(4) $$\frac{{\mathrm{dy}}_{c}}{\mathrm{dt}}=\frac{\partial y_{c}}{\partial t}+v.$$

Where the transition location has seasonality but returns to the same meridional position each year, the material derivative of the transition location is approximately equal to the local derivative, *∂*(*y*_*c*_)/*∂*(*t*), as long as the meridional velocity (*v*) is slow relative to the latitudinal shifts of the transition (*y*_*c*_) (see later for discussion on this latter assumption, as well as Supplement Text S3).

Here, we will use the latitude of the modeled transition of chlorophyll (the TZCF) (i.e., *c* = chl), *y*_chl_, as an example, but a similar process should be achievable for any biogeochemical transition. To build a mechanistic understanding of the controls on this transition and its movement, we write the time derivative of chlorophyll in terms of its driving variables. In light of the insight from the model in previous parts of the article (Fig. 7 and related discussion), we restate this equation in terms of total phosphorus, *P*_T_ and the ratio of chlorophyll to carbon in the phytoplankton, Θ, (Θ is affected by photoacclimation [Geider et al. 1998] and by changes in relative abundance of the different types of phytoplankton [Dutkiewicz et al. 2015]). Assuming that the spatial gradients in Θ are small (see discussion of this assumption below) allows us to write the following approximate differential equation for the time evolution of the chlorophyll transition (*see* Supplemental Text S4 for a more detailed derivation):

(5) $$\frac{{\mathrm{dy}}_{\mathrm{chl}}}{\mathrm{dt}}\approx -\frac{\partial y}{\partial P_{T}}\frac{\partial P_{T}}{\partial t}-\frac{\mathrm{chl}}{\Theta }\frac{\partial y}{\mathrm{\partial chl}}\frac{\partial \Theta }{\partial t}.$$

**Fig 8:**
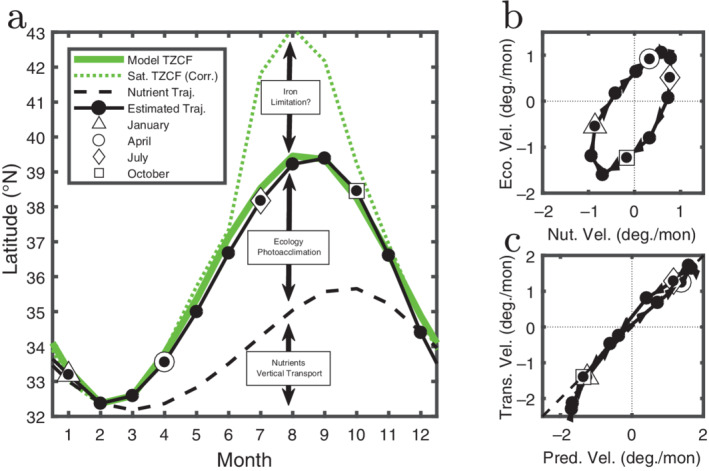
The seasonal motion of the TZCF along the 158 W transect (*see* Fig. 2) is compared with predictions. (**a**) The latitude of the Chl transition for satellite (dotted green line, corrected for the latitudinal shift in the Transition Zone between model and satellite) and model (solid green line) as calculated by the derivative method. The black solid line indicates the transition location as estimated by Eq. 5. Also shown is the nutrient driven component (dashed black line) as determined by the first term on the right‐hand side of Eq. 5. The difference between the two black lines is the ecologically driven component (second term on right hand side of Eq. 5). (**b**) The speed with which each of the two terms (nutrient vs. ecological) translate northward between each month plotted against one another. Open symbols indicate different months and link to those in panel (**a**). (**c**) The speed that the modeled transition moves northward [solid green line in (**a**)] vs. that estimated via the combination of terms in Eq. 5 [solid black line in (**a**)]. Dashed line is the one‐to‐one line, which would indicate that the estimated velocity was perfect.

From the model output, we can diagnose each component of Eq. 5. As such we integrate the right hand terms of Eq. 5 starting from February to provide an estimate for *y*_chl_(*t*) (solid black curve in Fig. 8a). The combination of the two components provide an estimate which is remarkably consistent with the actual modeled location of the TZCF (solid green curve). This is demonstrated quantitatively in Fig. 8c. The predicted phase velocity of the model TZCF agrees well with that produced using the two components in the right hand of Eq. 5. This correspondence provides confidence that the assumptions made above are reasonable: the latitudinal transport velocity (*v*) is small relative to the velocity of the TZCF and the spatial gradients in Θ are also small.

We now use the framework of Eq. 5 to help us understand the movement of the model TCZF. The first term on the right‐hand side of the equation represents the effect of changes in the total amount of nutrient in the surface ocean. Because of the transition from downwelling to upwelling, we expect the spatial derivative of *P*_T_ to be positive (i.e., higher *P*_T_ to the north), and the temporal derivative to be a function of mixed layer depth, and particle dynamics. The second term in the equation is a function of changes within the phytoplankton in terms of both biomass and chlorophyll content. The chlorophyll concentration, chl, at the transition is fixed by definition (*see* “Methods” section); the spatial derivative of chlorophyll is positive (i.e., higher chl to the north); and temporal changes in Θ are due to internal ecological/photo‐adaptive processes. Figure 8b shows the effects of composition (Ecological Velocity, right component in Eq. 5) and total nutrients (Nutrient Velocity, left component in Eq. 5) on the estimated phase velocity of the TZCF. In the late springtime as light increases and mixed layer depths start to decrease, *P*_T_ concentrations drop as particles sink out of the surface layer. At the same time, increasing light leads to a lower chlorophyll to carbon ratio (Θ) through photoacclimation. Additionally in the model, seasonal succession drives down the relative composition of diatoms (which are parameterized to have a higher maximum Θ; Dutkiewicz et al. 2015) leading to even lower Θ. In the fall and winter, deepening mixed layers bring *P*_T_ back to the surface as phosphate, which selects for diatoms which together with photoacclimation in all phytoplankton leads to higher Θ. Ecological and nutrient components of the phase velocity are therefore positive in the spring–summer and negative in the wintertime. In the model, we find that the internal processes (phytoplankton succession and photoadaptation) are equally important for driving the seasonal progression of the TZCF as changes in the bulk nutrient concentration. Figure 8a shows visually how the different processes affect the propagation of the TZCF. Starting in February, the trajectory of the TZCF can be split into its two components. The distance to the dashed curve, calculated by integrating forward the nutrient term in Eq. 5, is the portion of the excursion caused by vertical nutrient transport. The distance between the dashed and solid black curve is the portion due to ecological processes.

The above analysis provides a more thorough understanding of the controls on the movement of the TZCF in the model. Whether or not this is how the TZCF operates in the actual ocean remains an open question however. We plot the location of the satellite TZCF (dashed green curve in Fig. 8) corrected for the shift between the modeled and observed southern boundary of the Transition Zone. Here we have subtracted the shift in the mean location of the salinity transitions (0.94°) between model and satellite to more clearly compare the transition velocities. The model TZCF shows remarkable agreement with satellite observations except for July–September. This lends credence to the idea that the mechanisms driving the TZCF motion in the model from September through June are the same as in the real ocean. However, other mechanisms would be needed to explain the more rapid increase in the phase velocity of the satellite TZCF between June and July in the real world relative to the model. A likely explanation is that the model does not shift to iron limitation in these summer months/northern latitudes as is expected in the real ocean (Dutkiewicz et al. 2005; Boyd et al. 2007; Moore et al. 2013). Biogeochemical models in general still struggle to adequately model the iron cycle of the ocean (Tagliabue et al. 2016). The modeled chlorophyll is therefore likely artificially too high in the northern part of the Transition Zone, thus effectively blocking the propagation of the TZCF to high latitudes. Possibly other model limitations, including insufficient community complexity or inadequate parameterization of the chlorophyll dynamics, could also lead to the model/satellite discrepancies seen in the summer months. However, dictating these discrepancies in terms of transition phase velocities provides a unique opportunity to evaluate model against observations. More importantly, and especially for the months when model and observations of the TZCF are in close agreement, our framework provides a powerful method to explore the eco‐biogeochemical coupling in the surface ocean.

## Conclusion

Contextualizing the spatial–temporal structuring of the North Pacific in terms of the transitional boundaries of several physical, biogeochemical, and ecological fields allows for a systems level approach to understanding the Transition Zone. The magnitude and seasonal movement of these transitions are not determined by direct transport of matter nor are there strong physical barriers within the Transition Zone (Glover et al. 1994; Bograd et al. 2004; Ayers and Lozier 2010). Instead, the different transitions and their movement are set by the combination of: the seasonal physical forcings (heat, light) which lead to the repositioning of elements such as N and P between inorganic, living organic, and detrital matter; and the presence of climatological mean gradients.

As nutrient laden water steadily moves south in the North Pacific from high latitudes and into the subtropical gyre, a monotonic gradient in nutrient supply is formed. This monotonic gradient together with seasonal physical forcing (e.g., light, heat, mixed layer depth) and biological processes cause a range of sharp biogeochemical transitions that move seasonally, but not in a concerted way. Here, transitions are calculated as concentration contours which maximize the magnitude of the average meridional derivative. Our method explains the mechanistic underpinnings behind the previously empirical choice of 0.2 *μ*g L^−1^ as an indicator of the dynamics associated with the TZCF (Polovina et al. 2000). The differences in transitions for various biogeochemically relevant variables (e.g., Chl, diatom, photosynthesizing cyanobacteria) are linked through a complex interplay of biogeochemical couplings. We suggest a method to visualize (Figs. 4, 5) the movement of these transitions in terms of the two extreme types of physical transitions (salinity and temperature). The extreme transition types are seasonal or temperature‐like, and static or salt‐like transitions. Most biogeochemical and ecological variables, such as nutrients and chlorophyll, fall somewhere in‐between, demonstrating the nonlinear feedbacks and time lags due to biological processes.

For the modeled chlorophyll, we show explicitly how the movement of the transition (TZCF) can be explained with a partial differential equation in terms of the driving processes. These drivers are the strong gradient, the seasonal shift in the amount of total phosphorus, and the ecological shifts in phytoplankton community and photo‐physiology. Motion in early fall and late winter is controlled by nutrients while motion in early spring and late fall is due to ecological processes. Integrated over the entire year, both processes are of roughly equal importance with ecosystem shifts and photo‐adaptation accounting for a slight majority of the excursion.

The framework developed here also provides a method to compare different datasets (e.g., in situ vs. satellite product) and evaluate models. There are many reasons (including uncertainty in measurement methods) why the absolute value of any field (e.g., chlorophyll) will differ depending on the source (i.e., model, satellite, field observation). However, if the controlling mechanisms are the same, we would expect the behavior of their transitions (defined using a derivative criteria) to be similar, even if the absolute quantities differ. In terms of model evaluation, the framework provides an understanding of when important mechanisms are lacking in the model or model parameterizations are inadequate. Additionally, by reducing the problem to a set of transitions, data‐data or model‐data misfits become much clearer to understand. The method provides a path to parsimoniously compare different variable types across models, satellite, and in situ data.

Reducing the time variable concentration fields to a set of transitions provides a unique path toward understanding the North Pacific Transition Zone. Considering only the transitions, we can build simplified mechanistic models which predict their locations over time. Field campaigns could leverage these predictions to determine the best timing and routing for a cruise. The effect of physical processes is felt most strongly in February–March and leads to the biogeochemical transitions being close together nearer the southern boundary of the Transition Zone. In the spring and summer, biological processing leads to the northward propagation of these boundaries with a maximal northward extent and separation in late summer. Later in the year transitions have a southward propagation. Our mathematical expression of the TZCF comprises a few interpretable terms highlighting the key processes leading to these seasonal movements. Since the large‐scale biophysical drivers are common to all transitions, we are hopeful that future work can quantitatively tie their relative motion to specific biogeochemical/ecosystem processes. The framework of moving transitions provides a method to mechanistically understand the interplay between microbial dynamics, biogeochemical fluxes, and nutrient concentrations in the North Pacific.

## Conflict of Interest

None declared.

## Supporting information


**Appendix S1:** Supplementary Information
