# Supplementary material for: Moving ecological and biogeochemical transitions across the North Pacific
Source: Limnol Oceanogr. 2021 May 5;66(6):2442–54. doi: 10.1002/lno.11763 (PMC8252044; doi:10.1002/lno.11763)
Supplement: Supplementary file 1 — Appendix S1: Supplementary Information [file LNO-66-2442-s001.pdf]

Supplemental Information

S1: The Physical/Biogeochemical/Ecosystem Model

We employ a data constrained version of the MIT general circulation model (MITgcm, (Marshall et al. 1997)), ECCOv4, which is a state estimate based on satellite, float and in-situ observations of the ocean's physics over time (Forget et al. 2015). The state estimate has a nominally 1 degree horizontal resolution, and a set of vertical levels ranging from 10 m in the surface to 500 m in the deep ocean. A least-squares with Lagrange multipliers approach is used to obtain observationally adjusted initial and boundary conditions as well as internal physical model parameters (Forget et al. 2015). The biogeochemical/ecosystem model captures the cycling of C, N, P, Si and Fe as they pass through inorganic and (dead and living) organic pools. The ecosystem model equations and description of the parameterization and most parameter values are described in (Dutkiewicz et al. 2015) and (Dutkiewicz et al. 2020). The version used in this study resolves 35 phytoplankton (2 pico-prokaryotes, 2 pico-eukaryotes, 5 coccolithophores, 5 diazotrophs, 11 diatoms, 10 mixotrophic dinoflagellates) and 16 zooplankton, with size resolution spanning from 0.6 μm to 2500 μm Equivalent Spherical Diameter. Parameters influencing phytoplankton growth, grazing, and sinking are related to size (Ward et al. 2012; Dutkiewicz et al. 2020) with specific differences between the 6 functional groups (Dutkiewicz et al. 2020). Parameters for these are provided in (Dutkiewicz et al. 2020). Phytoplankton growth is limited by multiple nutrients (N, P, Fe, and Si in the case of diatoms), light (following (Geider et al. 1998)), and temperature (following (Kooijman 2000)). Grazing is parameterized using a Holling III function (Holling 1965) and is size-specific such that grazers can prey upon plankton 5 to 15 times smaller than themselves, with an optimal size of 10 times smaller. The emergent size distribution of the simulated plankton populations are strongly controlled both by the rate of supply of limiting nutrients (bottom up) and by grazing (top down) (Dutkiewicz et al. 2020).

The latitudinal distribution and seasonality of bulk ecosystem properties such as chlorophyll (see Main Text Fig. 2), phytoplankton biomass, nutrient concentrations, as well as distributions of size classes and functional groups are plausible in comparison with satellite and in-situ observations (Dutkiewicz et al. 2020). The biogeochemical component of the model has been employed in several recent studies (McParland and Levine 2019; Tréguer et al. 2018; Kuhn et al. 2019; Sonnewald et al. 2020), coupled to various physical models.

This version of the model was integrated for 20 years from 1992-2011. State variables and diagnostics include the biomass of the 51 plankton types, Chl-a, nutrient concentrations, temperature, salinity and velocity vectors. Here we use the 20-year monthly climatology of these variables, and focus on the seasonal cycle. Both temporal stochasticity due to synoptic forcing and unresolved mesoscale features can be important factors in this region, but a first order understanding at the larger spatial and seasonal times scales is essential before tackling these shorter/smaller scales.

The GitHub repository for the model configuration (https://github.com/CBIOMES/global-ocean-model), its latest DOI (http://doi.org/10.5281/zenodo.2653669) and its documentation (https://cbiomes.readthedocs.io/en/latest/index.html) are all readily available by following the links in parentheses.

S2: Satellite Data

Sea surface salinity (SSS) estimates were taken from SMAP (Fore et al. 2016) for years 2015-2019. Sea surface temperature (SST) data were taken from the GHRSST Level 4 AVHRR_OI Global Blended Sea Surface Temperature Analysis (GDS version 2) (Banzon et al. 2016) from the National Centers for Environmental Information (NCEI), available at the Physical Oceanography Distributed Active Archive Center (PODAAC) of the Jet Propulsion Laboratory from NASA, for years 1999-2019. Wind stress data are taken from the ASCAT scatterometer (Bentamy and Fillon 2012) from the NOAA ERDDAP data portal, for years 2009-2019. Chlorophyll data are from the merged Copernicus GlobColour product spanning 1997-2019 (including the SeaWiFS, MODIS-Squa, MERIS, VIIRSN and OLCI-S3A missions) (Garnesson et al. 2019). We consider monthly "climatologies" by using all available data averaged over the given years, and projecting onto a .5 degree grid.

S3: The Model Flow Field

In order to understand the biologically relevant transitions, it is important to understand the bulk meridional flow. Here we use results from the physical portion of the model, which is a data constrained state estimate of the ocean (Forget et al. 2015). Figure S1 shows the streamlines for the transect both averaged over the year (a) and across the season (b-e). The color represents the meridional component of the flow (in degrees/month) with cool colors being southward velocities and hot colors northward. Focusing on the seasonal average (S1a), we see that the average mixed layer flow within the Transition Zone is southward and its approximate boundaries (vertical solid and dashed lines) coincide with latitudinal changes in the average flow field. The model southern boundary (30-32 degrees north) and the northern boundary ($\sim$ 40 degrees north) are consistent with prior work (Roden 1998; Roden 1991). The northern most boundary of the Transition Zone marks a location of divergence in the subsurface portion of the latitudinal flow balanced by convergence in the near surface. With a net flow equatorward, water from the northern side moves south past this boundary, but not back. At the southern boundary of the Transition Zone we find strong convergence at the very surface, but the vertically integrated (to the base of the maximal mixed layer) flow remains equatorial. This allows water to enter the gyre from the north.

It is important to realize that water is transported rapidly between streamlines inside the mixed layer. When vertical gradients in velocity inside the mixed layer are strong it suggests a strong latitudinal mixing in that region. This condition holds both north and south of the Transition Zone, while inside it the flow is entirely equatorial. This process mixes water meridionally both south and north of the Transition Zone region. Inside the Transition Zone water moves continually southward.

Large seasonal oscillations in the flow are observable in Fig. S1b-e, yet they do not have a large effect on the seasonal motion of the biogeochemical transitions. The maximal meridional flow speeds attained in the model climatologies are $\sim$ 1 deg./month inside the Transition Zone. These values are attained at only the very surface. As water is continuously homogenized over the mixed layer, the effective meridional flow is much less: In the model climatology, we find it to be $\sim$ 3 deg./year. This coincides with the very weak meridional oscillation in the Transition Zone boundaries as defined by salinity ($\sim$ 2 deg./year). The

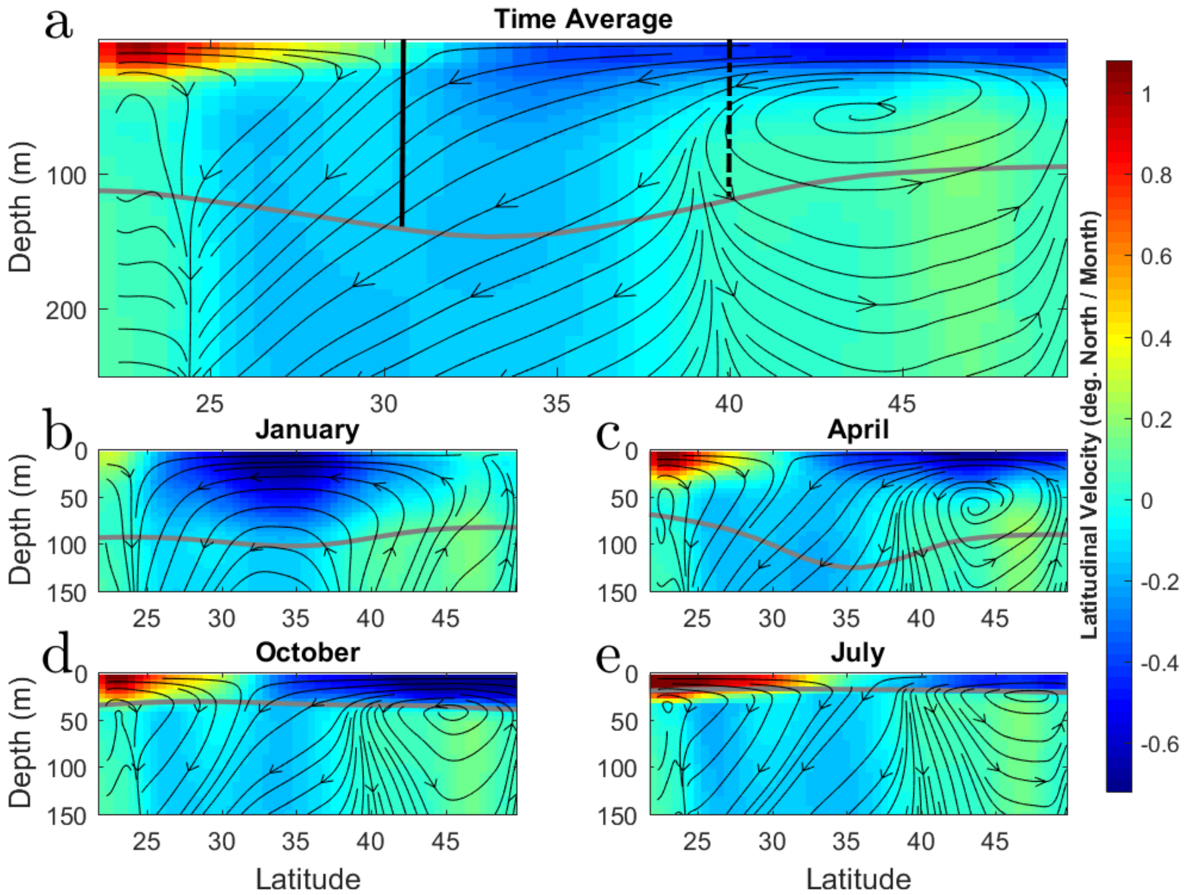

Figure S1: Time Variability and the Steady State Flow Field along 158W (transect shown in Main Text Fig. 2): These five panels are section data from the model climatologies showing both the annual and the time variability of the meridional and depth components of the velocity fields. Color represents the meridional component of the velocity field and the lines are the streamlines which incorporate the vertical and meridional component. Vertical solid and dashed lines are the approximate locations of the southern and northern boundaries of the Transition Zone. The dark gray line is the mixed layer depth (panels b-e) and its maximal depth over the year (panel a). Panels represent (a) The annual mean flow, (b) January, (c) April, (d) October, and (e) July.

most important aspect of the flow field is thus its time average. The rapid seasonal motion of biogeochemical transitions is not due to a Lagrangian oscillation in the surface waters.

S4: Derivation of equation used to examine phase velocity of Chl-a transition (Main Text Eq. 5)

The observed phase velocity (latitudinal shift) of the transition of any field, c, dy_c/dt, can be expressed as a sum of the phase velocity due only to forcings and the flow transport velocity, v, of that field:

$$\frac{dy_c}{dt} = \frac{\partial y_c}{\partial t} + v. \tag{s1}$$

Using $\langle\rangle$ to denote the yearly average and $'$ to denote the time dependent portion we can expand our equation s1,

$$\frac{dy_c}{dt} = \frac{\partial y_c'}{\partial t} + \langle\frac{\partial y_c}{\partial t}\rangle + \langle v\rangle + v'. \tag{s2}$$

For our transitions which return to the same latitude each year, the average phase velocity cancels out the average flow velocity, and we are left with an equation for the observed trajectory in terms of the time dependent parts of the phase velocity and flow,

$$\frac{dy_c}{dt} = \frac{\partial y_c'}{\partial t} + v'. \tag{s3}$$

Here, we will use the latitude of the modeled transition of chlorophyll (the TZCF) (i.e. $c = chl$), y_{chl}, as an example, but a similar process should be achievable for any biogeochemical transition. First we note that the time dependent portion of the transport v' is slow relative to the observed phase velocity of the TZCF, and as such, we can neglect this term (see later for discussion of this assumption). In this case:

$$\frac{dy_{chl}}{dt} \approx \frac{\partial y_{chl}'}{\partial t}. \tag{s4}$$

The observed phase velocity of the transition, dy_{chl}/dt, now depends only on latitude y, time t, and chlorophyll concentration, chl. Expanding in terms of the partial derivatives then gives us

$$\frac{dy_{chl}}{dt} \approx -\left(\frac{\partial chl}{\partial y}\right)^{-1} \frac{\partial chl}{\partial t}. \tag{s5}$$

This is the core equation which relates the phase velocity of the transition to both the background latitudinal gradients and the local, temporal processes. To build a mechanistic understanding of the controls on this transition and its movement, we write the time derivative of chlorophyll in terms of its driving variables. In light of the insight from the model in previous parts of the manuscript, we restate this equation in terms of total phosphorus, P_T, as well as the total carbon biomass of phytoplankton, ρ (importantly it will not be in the final equation), and the ratio of chlorophyll to carbon in the phytoplankton, Θ, where $\rho * \Theta = chl$, (Θ is affected by photoacclimation and by changes in relative abundance of the different types of phytoplankton (Dutkiewicz et al. 2015)):

$$\frac{dy_{chl}}{dt} \approx -\left(\frac{\partial chl}{\partial y}\right)^{-1} \left(\Theta \frac{\partial \rho}{\partial P_T}\frac{\partial P_T}{\partial t} + \rho\frac{\partial \Theta}{\partial t}\right). \tag{s6}$$

Expanding the second partial derivative on the right hand side in terms of the latitudinal derivative, and assuming that the spatial gradients in Θ are small (see discussion of this assumption below) allows us to write the following approximate differential equation for the time evolution of the chlorophyll transition

$$\frac{dy_{chl}}{dt} \approx -\frac{\partial y}{\partial P_T}\frac{\partial P_T}{\partial t} - \frac{chl}{\Theta}\frac{\partial y}{\partial chl}\frac{\partial \Theta}{\partial t}. \tag{s7}$$

From the model output, we can diagnose each component of Equation s7. As such we integrate the right hand terms of Equation s7 starting from February to provide an estimate for $y_{chl}(t)$ (solid black curve in Main Text Fig. 8a). The combination of the two components provide an estimate which is remarkably consistent with the the actual modeled location of the TZCF (solid green curve). This can be seen more quantitatively in the lower left panel in the same figure. The predicted phase velocity of the model TZCF agrees well with that produced using the two components in the right hand of Equation s7. This correspondence provides confidence that the assumptions made above are reasonable: the latitudinal transport velocity (v') is small relative to the velocity of the TZCF and the spatial gradients in Θ are small.

References

Banzon, Viva, Thomas M Smith, Toshio Mike Chin, Chunying Liu, and William Hankins. 2016. A long-term record of blended satellite and in situ sea-surface temperature for climate monitoring, modeling and environmental studies. Earth System Science Data 8.1, pp. 165–176.

Bentamy, Abderrahim and Denis Croize Fillon. 2012. Gridded surface wind fields from Metop/ASCAT measurements. International journal of remote sensing 33.6, pp. 1729–1754.

Dutkiewicz, S, A. E. Hickman, O Jahn, W. W. Gregg, C. B. Mouw, and M. J. Follows. 2015. Capturing optically important constituents and properties in a marine biogeochemical and ecosystem model. Biogeosciences 12.14, pp. 4447–4481.

Dutkiewicz, Stephanie, Pedro Cermeno, Oliver Jahn, Michael J Follows, Anna E Hickman, Darcy AA Taniguchi, and Ben A Ward. 2020. Dimensions of marine phytoplankton diversity. Biogeosciences 17.3, pp. 609–634.

Fore, Alexander G, Simon H Yueh, Wenqing Tang, Bryan W Stiles, and Akiko K Hayashi. 2016. Combined active/passive retrievals of ocean vector wind and sea surface salinity with SMAP. IEEE Transactions on Geoscience and Remote Sensing 54.12, pp. 7396–7404.

Forget, Gael, J. M. Campin, Patrick Heimbach, Christopher N. Hill, and Carl Ponte R. M. Wunsch. 2015. ECCO version 4: An integrated framework for non-linear inverse modeling and global ocean state estimation. Geoscientific Model Development 8.10, pp. 3071–3104.

Garnesson, Philippe, Antoine Mangin, Odile Fanton d'Andon, Julien Demaria, and Marine Bretagnon. 2019. The CMEMS GlobColour chlorophyll a product based on satellite observation: multi-sensor merging and flagging strategies. Ocean Science 15.3, pp. 819–830.

Geider, Richard J., Hugh L. MacIntyre, and Todd M. Kana. 1998. A dynamic regulatory model of phytoplanktonic acclimation to light, nutrients, and temperature. Limnology and Oceanography 43.4, pp. 679–694.

Holling, C. S. 1965. The Functional Response of Predators to Prey Density and its Role in Mimicry and Population Regulation. Memoirs of the Entomological Society of Canada 97.S45, pp. 5–60.

Kooijman, S. A. L. M. 2000. *Dynamic Energy and Mass Budgets in Biological Systems.* Cambridge University Press.

Kuhn, A. M., S. Dutkiewicz, O. Jahn, S. Clayton, T. A. Rynearson, M. R. Mazloff, and A. D. Barton. 2019. Temporal and Spatial Scales of Correlation in Marine Phytoplankton Communities. Journal of Geophysical Research: Oceans 124.12, pp. 9417–9438.

Marshall, John, Alistair Adcroft, Chris Hill, Lev Perelman, and Curt Heisey. 1997. A finite-volume, incompressible navier stokes model for, studies of the ocean on parallel computers. Journal of Geophysical Research C: Oceans 102.C3, pp. 5753–5766.

McParland, Erin L. and Naomi M. Levine. 2019. The role of differential DMSP production and community composition in predicting variability of global surface DMSP concentrations. Limnology and Oceanography 64.2, pp. 757–773.

Roden, G I. 1991. Subarctic-subtropical transition zone of the North Pacific: Large-scale aspects and mesoscale structure. NOAA Technical Report NMFS 105, pp. 1–38.

Roden, Gunnar I. 1998. Upper ocean thermohaline, oxygen, nutrient, and flow structure near the date line in the summer of 1993. Journal of Geophysical Research C: Oceans 103.3336, pp. 12919–12939.

Sonnewald, Maike, Maike Sonnewald, Stephanie Dutkiewicz, Christopher Hill, and Gael Forget. 2020. Elucidating ecological complexity: Unsupervised learning determines global marine eco-provinces. Science Advances 6.22, eaay4740.

Tréguer, Paul and others. 2018. Influence of diatom diversity on the ocean biological carbon pump. Nature Geoscience 11.1, pp. 27–37.

Ward, B. A., S. Dutkiewicz, O. Jahn, and M. J. Follows. 2012. A size-structured food-web model for the global ocean. Limnology and Oceanography 57.6, pp. 1877–1891.
